# Refractory dermatitis contributed by pityriasis versicolor: a case report

**DOI:** 10.1186/s13256-021-02818-1

**Published:** 2021-04-23

**Authors:** Mingjia Li, Natalie Spaccarelli, Kari Kendra, Richard C. Wu, Claire Verschraegen

**Affiliations:** 1 Division of Hospital Medicine, The Ohio State University Comprehensive Cancer Center, Starling Loving Hall, 320 W. 10th Ave, Columbus, Ohio 43210 USA; 2 Division of Dermatology, The Ohio State University Comprehensive Cancer Center, 395 W 12th Ave, Columbus, Ohio 43210 USA; 3 Division of Medical Oncology, The Ohio State University Comprehensive Cancer Center, Lincoln Tower 1300, 1800 Cannon Dr, Columbus, Ohio 43210 USA

**Keywords:** Immune-related adverse event, Skin infection, Dermatitis, Pityriasis versicolor

## Abstract

**Background:**

Dermatologic toxicity is a very common immune-related adverse event (irAE) for patients with melanoma who are receiving immune checkpoint inhibitor therapy (ICI). Concurrent skin infection, such as in the case of pityriasis versicolor reported here, can mimic and/or exacerbate dermatologic toxicity from irAE.

**Case presentation:**

A 58-year-old Caucasian man with a history of pityriasis versicolor infection and metastatic melanoma received ICI therapy. He developed progressively worsening pruritic maculopapular lesions 22 weeks into his treatment that ultimately covered 40% of his body. He was diagnosed with dermatologic toxicity due to ICI therapy with concurrent pityriasis versicolor. He was initially started on topical steroid and topical antifungal cream but achieved minimum improvement. His treatment was then escalated to oral prednisone, but it only achieved modest control of his dermatitis. All subsequent attempts to wean him from oral prednisone resulted in worsening of his dermatitis. Eventually he was started on oral fluconazole in combination with prednisone, which led to rapid resolution of his dermatitis.

**Conclusion:**

We report a case of dermatological toxicity due to an irAE with concurrent pityriasis versicolor. The steroid treatment for irAE was likely exacerbating the underlying fungal infection, and the fungal infection was in term mimicking the symptoms of irAE. This patient’s severe dermatitis was only brought under control after receiving a more potent antifungal therapy in combination with a steroid. It is vital to look beyond the irAE when managing dermatitis in patients receiving ICI therapy.

## Background

Immune checkpoint inhibitors (ICI) have led to a remarkable improvement in the long-term survival of patients with melanoma and other types of cancer [1, 2]. However, the prevalence of ICI usage has led to a surge in a number of dermatologic toxicities due to immune-related adverse events (irAEs) [3]. For some patients, these irAEs have caused a considerable amount of morbidity and mortality [3]. Aside from frequent dermatologic toxicities, patients with irAEs frequently require treatment with immunosuppressive agents that can increase their risk for opportunistic skin infections [4]. Here we report a case of refractory dermatitis due to an irAE with associated underlying pityriasis versicolor.

### Case presentation

**Fig. 1:**
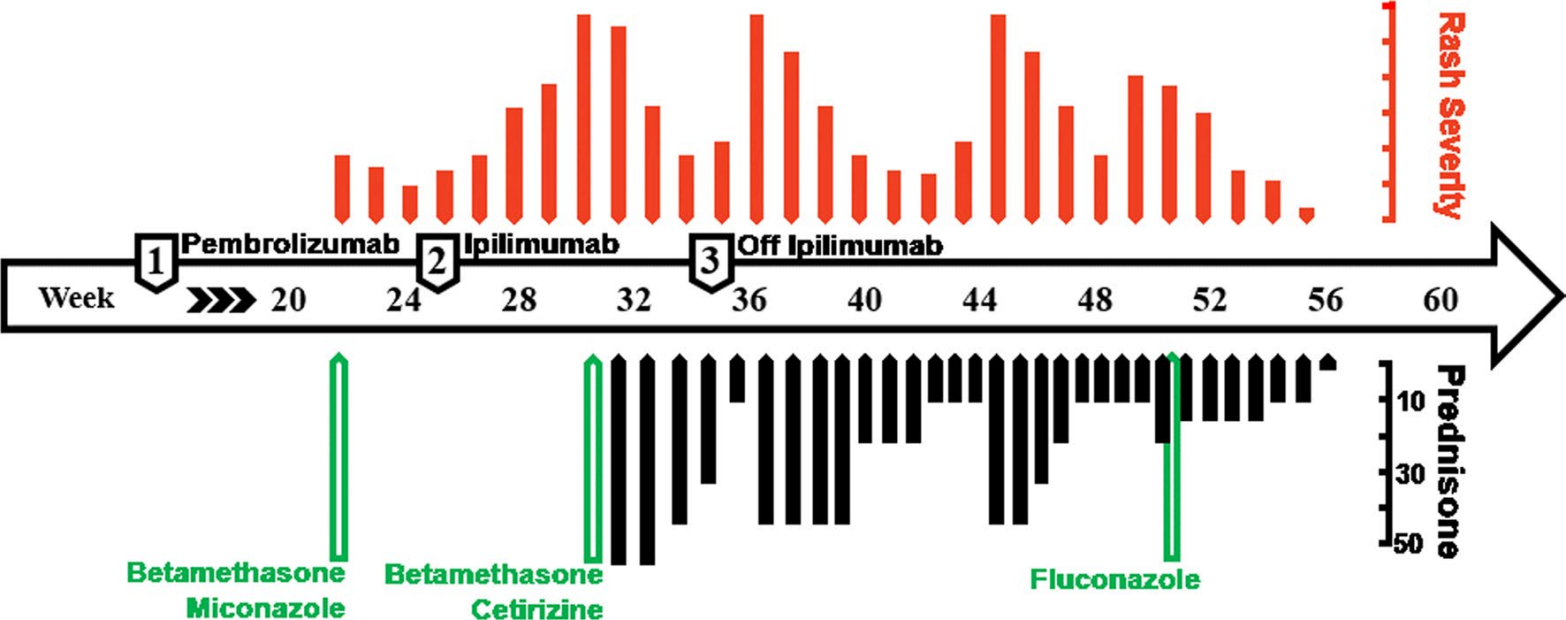
Time-line showing the severity of the dermatitis (top, red bars) over time, and treatment with prednisone in milligrams (bottom, black bars) and other treatments (green bars).

**Fig. 2:**
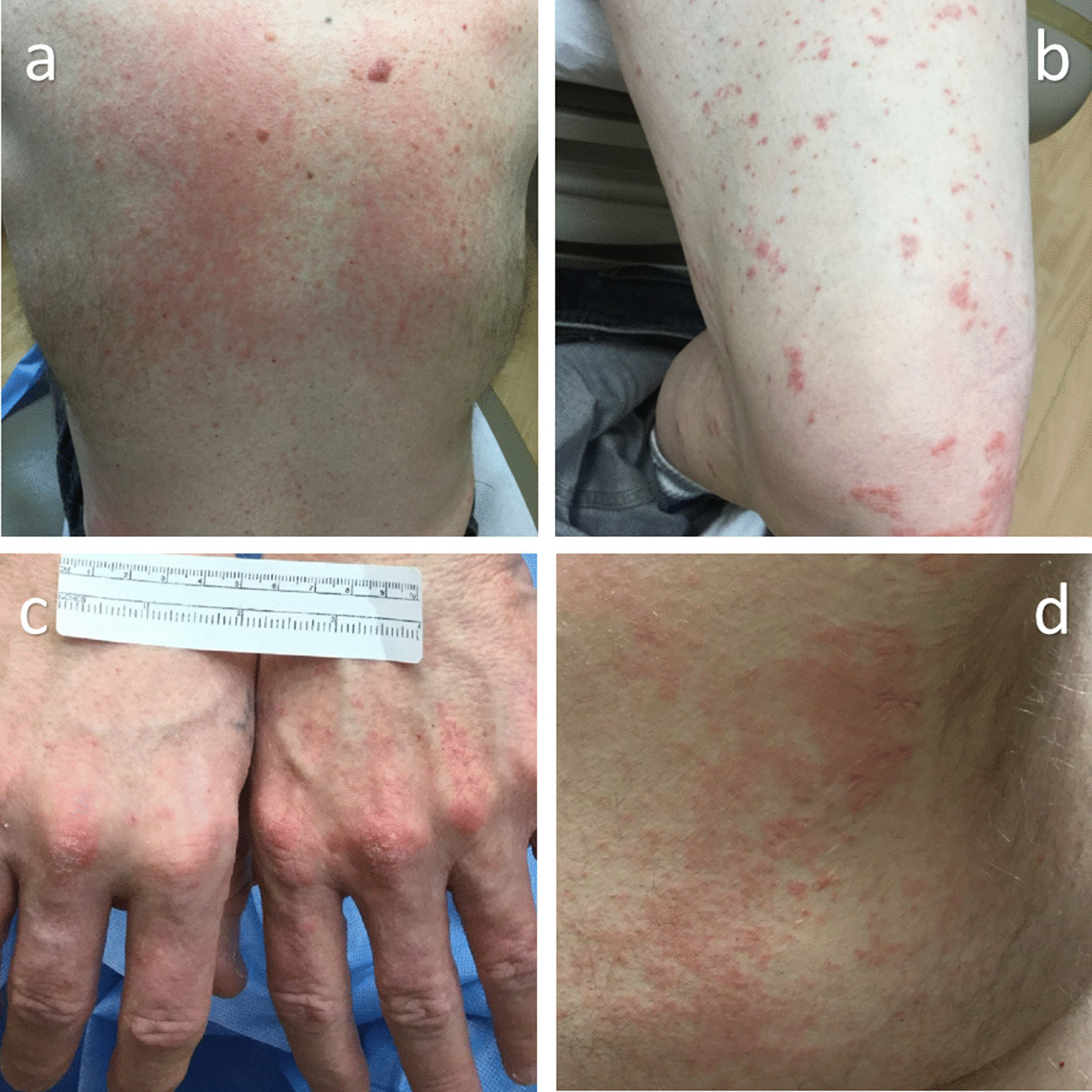
Initial presentation of dermatitis involving the back(**a**), lower extremity (**b**), hands (**c**), and right lateral torso (**d**)

A 58-year-old Caucasian man with a history of pityriasis versicolor and metastatic melanoma was started on pembrolizumab 200 mg every 21 days. He developed pruritic maculopapular rashes throughout his body 22 weeks into the treatment course. In some areas, the maculopapular lesions coalesced into patches and plaques. Many of these lesions were scaly in appearance and resembled his prior pityriasis versicolor infection. The skin eruptions were mainly in the abdomen, back, and extremities (Figs. 1, 2). He was started on topical betamethasone diproprionate 0.05% and miconazole nitrate 2% cream for the dermatitis due to the irAE with associated underlying pityriasis versicolor. The skin lesions improved with these topical therapies.

**Fig. 3:**
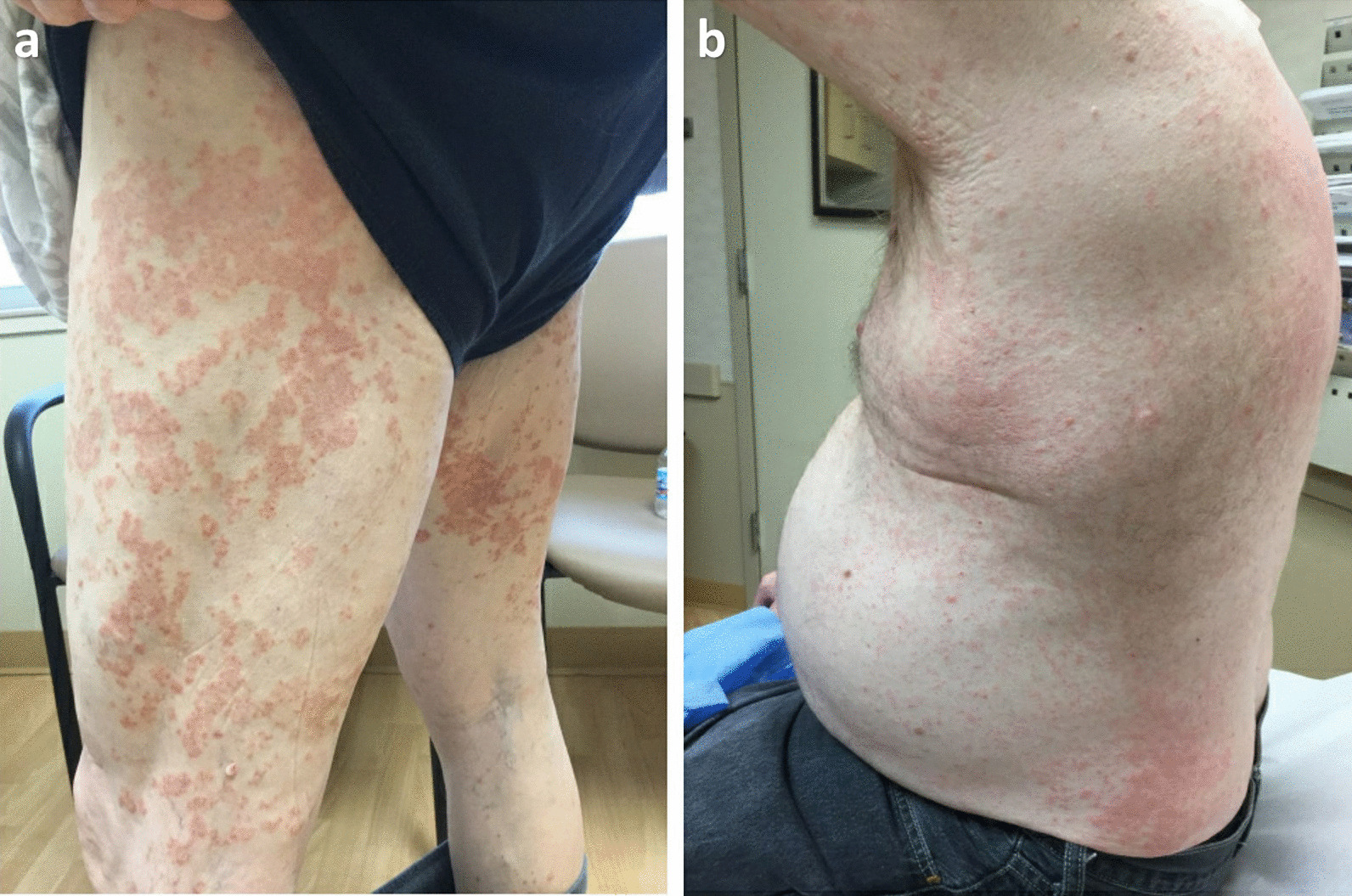
Worsening of dermatitis after his initial presentation in the lower extremities (**a**) and torso (**b**)

By 25 weeks after starting pembrolizumab, his melanoma then progressed and the immunotherapy regimen was switched to ipilimumab 3 mg/kg every 21 days. Five weeks later, he had a worsening of the dermatitis with severe pruritus. The dermatitis covered approximately 40% of his body surface area and involved his torso, bilateral hands, and lower extremities (Fig. 3). A second course of topical betamethasone diproprionate 0.05% cream, daily cetirizine 10 mg tablet, and miconazole nitrate 2% cream was administered to the patient, without improvement. Oral prednisone 50 mg (0.5 mg/kg) daily was then prescribed, and the extent of his rashes from dermatitis improved.

Subsequent multiple attempts to wean him from the prednisone resulted in rapid worsening of his skin condition. His melanoma disease burden continued to worsen and ipilimumab was discontinued, although the dermatitis did not abate. His diffuse rashes were only moderately controlled with prednisone and topical antifungal cream. Due to the persistent refractory dermatitis, which resembled his prior pityriasis versicolor eruptions, the decision was taken to treat the dermatitis with a stronger oral antifungal therapy for pityriasis versicolor while continuing prednisone for the dermatologic toxicity due to the irAE. He was started on fluconazole 200 mg daily on day 1 followed by 100 mg daily for 6 additional days while continuing with a prednisone taper at 15 mg daily. A skin biopsy of the flank torso was also performed on the day of starting fluconazole and showed superficial middle dermal perivascular dermatitis with associated spongiosis. The pathology was compatible with hypersensitivity from either medication or other potential allergens. The rashes resolved within days after starting fluconazole without any reported intolerability to the treatment, and the patient came off the prednisone shortly without the need of any further escalation.

His performance status remained optimal, and his treatment was switched to temozolomide 5 weeks later given optimal performance status. Unfortunately his cancer progressed further, and he was rechallenged with nivolumab 1 year later. No significant dermatological toxicity was noted.

## Discussion

As the indications for ICI are expanding at a rapid pace, we are seeing an increasing number of patients with moderate to severe irAEs. Approximately 30–40% of patients who are treated with programmed cell death protein 1 (PD-1) blockers, such as nivolumab or pembrolizumab, develop dermatologic complications [3, 5]. More severe dermatological incidents are seen in patients treated with cytotoxic T-lymphocyte-associated protein 4 (CTLA-4) inhibitors, such as ipilimumab, and significantly worse irAEs are often seen with the combination of PD-1/CTLA-4 inhibitors [3].

Many oncologists have adapted the grading system of the Common Terminology Criteria for Adverse Events Version 5 (CTCAE v5) to quantify the severity of irAEs, and a myriad of organizations have designed protocols in an attempt to streamline the diagnosis and treatment of dermatologic irAEs [6, 7]. Current guidelines recommend a thorough diagnostic workup to rule out other potential etiologies, such as infections, reactions from another medications, and systemic diseases [7]. A consultation with an experienced dermatologist familiar in managing dermatologic irAEs is highly recommended for CTCAE grade 3 or higher skin toxicity before resuming ICI [6, 7].

In addition to the dermatologic toxicity due to the irAE, this patient had an underlying pityriasis versicolor. Pityriasis versicolor is a very common fungal infection in tropical climates and a less frequently occurring one in other climate zones. It is caused by *Malassezia* species, which is a skin flora saprophyte [8]. The exact mechanism of pathogenesis is unknown, but the immunosuppressed patients have an increased risk of developing pityriasis versicolor [4]. The skin lesions can appear in multiple forms, ranging from mildly erythematous to hypopigmented forms, and sometimes hyperpigmented ones. In the advanced form, these small lesions can become confluent [8]. Most patients with pityriasis versicolor are asymptomatic and only experience cosmetic changes of their skin [8]. However, in patients who are receiving oral corticosteroid or who are immunocompromised, these infections can be severe [8]. Patients with pityriasis versicolor are usually diagnosed by physical examation. Light microscopy observation of a potassium hydroxide preparation can be used for confirmation [8]. Skin infection due to pityriasis versicolor generally responds well to topical antifungal agents [9]. The typical duration of treatment ranges from 1 to 4 weeks [9]. Oral antifungal agents, such ketoconazole, itraconazole, or fluconazole, are reserved for florid infection or recurrent disease [9].

Our patient had dermatitis due to an irAE from pembrolizumab and ipilimumab. In addition, he had pityriasis versicolor that predated the immunotherapy and which was exacerbated by the treatment for the irAE. His underlying fungal infection was mimicking features of the dermatologic toxicity due to the irAE. Although the pityriasis versicolor was diagnosed based on the clinical features and no yeast or hyphae were seen on biopsy, the rapid resolution of his dermatitis after a short course of intensified antifungal therapy indirectly confirmed our diagnosis. We suspected that the chronic topical antifungal and steroid cream might have contributed to a false negative biopsy result. Given that his rash was already resolved, we did not perform any additional fungal testing. Furthermore, no significant skin toxicity was noted when he was rechallenged with nivolumab.

In this case, it is possible that fluconazole, a CYP3A4 inhibitor, could potentially have increased the serum concentration of prednisone due to drug–drug interactions [10]. However, our patient was only receiving a very low dose of prednisone when the treatment with the low dose of fluconazole was started. The complete resolution of his skin condition could not be adequately explained by a small to moderate increase in steroid concentration alone. In addition, he did not require any additional escalation of steroid dose after completing the 7-day course of fluconazole. His overall response to treatment was consistent with the dermatologic toxicity due to an irAE with an associated underlying fungal infection.

## Conclusion

Immunotherapy is standard of care for many cancers. Although treated patients frequently develop dermatitis as a part of irAEs, one must remain vigilant and consider other potential differential diagnoses other than irAEs when managing ICI-related dermatologic complications.

## Data Availability

In accordance with local and/or U.S. Government laws and regulations, any materials and de-identified data that are reasonably requested by others will be made available in a timely fashion.

## Abbreviations

CTLA-4: Cytotoxic T-lymphocyte-associated protein 4
ICI: Immune checkpoint inhibitors
irAEs: Immune-related adverse events
PD-1: Programmed cell death protein 1
